# circFOXO3 Induced by KLF16 Modulates Clear Cell Renal Cell Carcinoma Growth and Natural Killer Cell Cytotoxic Activity through Sponging miR-29a-3p and miR-122-5p

**DOI:** 10.1155/2022/6062236

**Published:** 2022-08-29

**Authors:** Fafen Yang, Yuke Chen, Linxue Luo, Shengbin Nong, Tong Li

**Affiliations:** ^1^Department of Nephrology, Affiliated Hospital of Youjiang Medical University for Nationalities, Guangxi, China; ^2^Nursing Department, Affiliated Hospital of Youjiang Medical University for Nationalities, Guangxi, China

## Abstract

Renal cell carcinoma (RCC) is one of the most common urological malignancies with high incidence and metastatic relapse. Clear cell RCC (ccRCC) comprises nearly 70% of all RCC cases and is responsible for the majority of morbidity and mortality of RCC. Due to the poor diagnosis strategy and unsatisfactory clinical intervention, ccRCC causes a huge economic burden and poor patient quality of life; therefore, novel diagnostic or therapeutic targets for ccRCC are urgently needed. This study investigated the biological role of circFOXO3 in ccRCC development, showing that circFOXO3 is highly expressed in RCC cells and tissues and inhibits the viability of ccRCC cells. circFOXO3 dysregulation regulates NK cell cytotoxicity towards RCC cells by directly sponging miR-29a-3p and miR-122-5p. Overexpression of miR-29a-3p or miR-122-5p attenuated NK cell toxicity towards RCC cells and the transcriptional factor Kruppel-Like Factor 16 (KLF16) regulates circFOXO3 expression in RCC cells. In conclusion, this study has partially elucidated the function of circFOXO3 in ccRCC development, providing potential novel therapeutic targets for ccRCC.

## 1. Introduction

Renal cell carcinoma (RCC), which originates from the renal epithelium, is a common urological tumor accounting for approximately 90% of kidney cancers [1]. RCC is well known for its high incidence and metastatic relapse [2] with clear cell RCC (ccRCC) responsible for 70% of RCC cases, as well as the primary morbidity and mortality of RCC [3, 4]. Surgical resection remains the primary clinical intervention of ccRCC because it is resistant to chemotherapy or radiotherapy; however, approximately 30% of ccRCC patients eventually develop metastasis [5, 6]. The molecular mechanisms that contribute to ccRCC initiation or progression have been widely investigated but the mechanisms of ccRCC development remain poorly understood.

Circular RNAs (circRNAs) are a newly identified class of noncoding RNAs spliced from exons or introns and feature a covalently closed loop structure [7, 8]. The specific structure without terminal 5′ caps and 3′ poly-A tails means that circRNAs are abundant, conserved, and endogenous RNAs in mammalian cells [9]. With the technological innovation of high-throughput sequencing, the molecular characterization and biological functions of circRNAs have been studied in depth, including cancer initiation and development [10, 11], for example, the role of circRNAs in hepatocellular carcinoma, breast cancer [12], head and neck carcinoma, and gastric cancer [13–16]. In the past five years, several circRNAs have been studied in ccRCC development. Wang et al. demonstrated that circHIAT1 functions in ccRCC development through miR-195-5p/29a-3p/29c-3p/CDC42 signalling [17], while Xue et al. elucidated that circ-AKT3 suppresses ccRCC progression by altering the miR-296-3p/E-cadherin pathway [18]. Han et al. explored the aggravative effect of circATP2B1 in ccRCC progression [19], and Lv et al. revealed that circAGAP1 promotes ccRCC tumorigenesis via acting as a sponge for miR-15-5p [20]. Thus, there is increasing evidence that circRNAs play vital roles in ccRCC progression, so they are a promising direction for ccRCC basic research.

circRNA forkhead box O3 (circFOXO3), also known as hsa_circ_0006404, is derived from exon 2 of the FOXO3 gene [21]. It has been demonstrated to be involved in multiple cancers [21–25], but the role of circFOXO3 in ccRCC development remains unclear.

This study hypothesized that circFOXO3 plays a role in the development of ccRCC and showed that circFOXO3 was highly expressed in ccRCC tumor tissues and cells. Furthermore, circFOXO3 inhibited ccRCC cell proliferation and regulated NK cell-mediated cytotoxicity towards ccRCC cells by directly targeting miR-29a-3p and miR-122-5p. Moreover, circFOXO3 expression in ccRCC cells was transcriptionally mediated by KLF16. Our study identified a novel KLF16/circFOXO3/miR-29a-3p/miR-122-5p signalling pathway in ccRCC progression which might be a new direction for ccRCC basic research.

## 2. Materials and Methods

### 2.1. Human Tissue Samples

Thirty pairs of ccRCC tumor tissues and their adjacent normal tissues were collected from patients who had not undergone radiotherapy or chemotherapy at the Affiliated Hospital of Youjiang Medical University for Nationalities. All patients provided informed consent, and the study was approved by the ethics committee of the Affiliated Hospital of Youjiang Medical University for Nationalities (YYFY-LL-2021-35). After surgery, all specimens were immediately stored at -80°C for further analysis and all tissue samples were diagnosed and confirmed by three pathologists independently.

### 2.2. Cell Culture and Transfection

Human RCC cell lines (ACHN, A498, 786-O, 769-P, and Caki-1) and the normal renal cell HK-2 were obtained from the American Type Culture Collection (Manassas, VA, USA) and cultured in high-glucose DMEM (Gibco, CA, USA) with 10% FBS (Gibco) at 37°C and 5% CO_2_. The short hairpin RNA (shRNA) was applied to silence circFOXO3 levels using the Pglvu6/Puro vector, with the full length of the circFOXO3 coding sequence amplified and cloned into a pcDNA3.1 vector for overexpression. miR-29a-3p and miR-122-5p mimics, small interfering RNA targeting KLF16, and the DNA fragment encoding the mutant circFOXO3 were synthesized and purchased from VectorBuilder (Guangzhou, China). A Lipofectamine 3000 kit (Invitrogen, CA, USA) was used to perform transfections following the manufacturer's protocol.

### 2.3. qRT-PCR and RNase R Treatment

All RNAs in this study were isolated from tissues and cells using Trizol reagent (Invitrogen). A PrimeScript™ RT kit (Takara, China) was used to reverse transcribe RNA into cDNA. A MicroRNA Reverse Transcription Kit (Takara Biotechnology, Japan) was applied to accomplish miRNA reverse transcription, with qRT-PCR for RNA detection performed on an ABI 7500 Fast PCR System (Carlsbad, CA, USA) using an SYBR Green PCR Master Mix (TOYOBO, Japan). GAPDH and U6 were applied as the internal control, and RNA expression was analyzed via the 2–*ΔΔ*CT method. RNase R treatment was performed by adding 1 unit RNase R to 1 *μ*g of RNA for 20 min at room temperature. The primers used were as follows: circFOXO3 forward: 5′-GTGGGGAACTTCACTGGTGCTAAG-3′, circFOXO3 reverse: 5′-GGGTTGATGATCCACCAAGAGCTCTT-3′, FOXO3 forward: 5′-ACATGGGCTTGAGTGAGTCC-3′, FOXO3 reverse: 5′-GCCTGAGAGAGAGTCCGAGA-3′, miR-29a-3p-RT: 5′-GTCGTATCCAGTGCAGGGTCCGAGGTGCACTGGATACGACC TGAACAC-3′, miR-29a-3p forward: 5′-TGCGGACTGATTTCTTTTGG-3′, miR-29a-3p reverse: 5′-CCAGTGCAGGGTCCGAGGT-3′, miR-122-5p-RT: 5′-UGGAGUGUGACAAUGGUGUUUG-3′, miR-122-5p forward: 5′-GGGTGGAGTGTGACAATGG-3′, miR-122-5p reverse: 5′-CAGTGCGTGTCGTGGAGT-3′, U6-RT: 5′-GTCGTATCCAGTGCAGGGTCCGAGG TGCACTGGATACGACAAAATATGGAAC-3′, U6 forward: 5′-TGCGGGTGCTCGCTTCGGCA GC-3′, U6 reverse: 5′-CCAGTGCAGGGTCCGAGGT-3′, GAPDH forward: 5′-GGGAAACTGTGGC GTGAT-3′, and GAPDH reverse: 5′-GTGGTCGTTGAGGGCAAT-3′.

### 2.4. Fluorescence In Situ Hybridization (FISH) Assay

Fluor 488-labelled probes to detect circFOXO3 were designed and synthesized by RiboBio. Cells were subjected to prehybridization buffer for fixation, and the probes were hybridized with the cells hybridized for 120 min at 50°C. DAPI was used to stain the nuclei, and a FISH Kit (RiboBio, Guangzhou, China) was used to detect probe signals, with the images visualized and captured by a Leica SP8 laser scanning confocal microscope.

### 2.5. Cell Proliferation

The proliferation of ACHN and Caki-1 cells was quantified by ethynyldeoxyuridine (EdU) incorporation using a Cell-Light EdU DNA Cell Proliferation Kit (RiboBio, Guangzhou, China) according to the manufacturer's protocol. First, cells were cultured at 37°C, 5% CO_2_ for two days. Subsequently, the cells were incubated with 50 mM EdU solution for 120 min, fixed with 4% paraformaldehyde, and stained with Apollo Dye Solution and DAPI. The results were analyzed by ImageJ software (National Institutes of Health, AZ, USA).

### 2.6. Apoptosis

The apoptosis rate of ACHN and Caki-1 cells was assessed using an Annexin V-PI apoptosis kit (AccuRef Scientific). Briefly, cells were incubated with EPI for 24 h and fixed with Annexin V-PI. After double staining, the apoptotic cells were detected using the FACSAria II flow cytometer (BD Biosciences) and calculated by FlowJo software (version 7; FlowJo LLC).

### 2.7. RNA Pull-Down Assay

Biotinylated circFOXO3 probes or their mutants were transfected into ACHN or Caki-1 cells, sequences as Bio-circFOXO3: ATGCAGTGACAGGTTGTGCCGGATGGAGTTCTGCTTTGCC, Bio-NC: GGCAAAGCAGAACTCCATCCGGCACAACCTGTCACTGCAT. C-1 magnetic beads (RiboBio, China) were lysed for 180 min at 4°C and then washed three times with wash buffer before qRT-PCR to detect the retrieved RNAs.

### 2.8. Luciferase Reporter Assay

circFoxo3 and the 3′-untranslated region (3′-UTR) wild or mutant fragments of miRNA plasmids or KLF16 and FOXO3 mutant region mimic fragments of the 3′-untranslated region (3′-UTR) wild or mutant plasmids were cotransfected into cells, respectively, using Lipofectamine 3000. After 48 h, a Dual-Luciferase Reporter Assay System (Promega) was used to measure the luciferase activities of both firefly and renilla luciferase.

### 2.9. NK Cell Purification and Expansion

K562 aAPC was incubated with human peripheral blood mononuclear cells purified from healthy donors in a 1 : 2 ratio after 100 Gy radiation. Subsequently, expanded NK cells were cultured for 2~3 weeks for further study. The experimental procedure was as previously described [26].

### 2.10. Calcein Release Assay

Cells were stained with 30 *μ*M calcein-AM (Dojindo) for 30 min at room temperature and cultured with NK cells at 20, 40, or 60 E/T ratios for 3 h before the supernatant was collected for fluorescent detection at 490 nm excitation. Subsequently, the spontaneous release value (SRV) and maximum release value (MRV) were measured to calculate [(test release value–SRV)/(MRV–SRV)].

### 2.11. Perforin Polarization Assay

RCC cells and NK cells were cocultured (1 : 1) for 30 min and then seeded onto poly-D-lysine-coated slides in a 12-well plate for 1 h at room temperature. The cells were fixed with 4% paraformaldehyde and permeabilized with 0.5% Triton X-100 in PBS before incubation with the primary antibody (antiperforin) and secondary antibody (Alexa Fluor 568-conjugated secondary antibody). The results were visualized by a confocal microscope, and the polarization was recorded.

### 2.12. Conjugation Assay

RCC cells were stained with 30 *μ*M calcein-AM (Dojindo) for 30 min at room temperature and then incubated with NK cells for 0, 20, 40, and 60 min. The NK cells were fixed with CFSE and ACHN or Caki-1 cells were fixed with CellTracker orange CMTMR. Subsequently, the mixed cells (ratio 1 : 1) were subjected to flow cytometry, and the proportion of conjugated NK cells was calculated as follows: [the double‐positive events]/[the total events].

### 2.13. Statistical Analysis

All data were analyzed using SPSS 21.0 (IBM, IL), and the results are presented as mean ± standard deviation (SD). Experiments were repeated at least three times. Two-way analysis of variance (ANOVA) or Student *t*-tests were used to calculate the significance among groups. *P* < 0.05 was considered statistically significant; ^∗^*P* < 0.05, ^∗∗^*P* < 0.01, and ^∗∗∗^*P* < 0.001.

## 3. Results

### 3.1. Expression and Characterization of circFOXO3 in ccRCC

**Figure 1:**
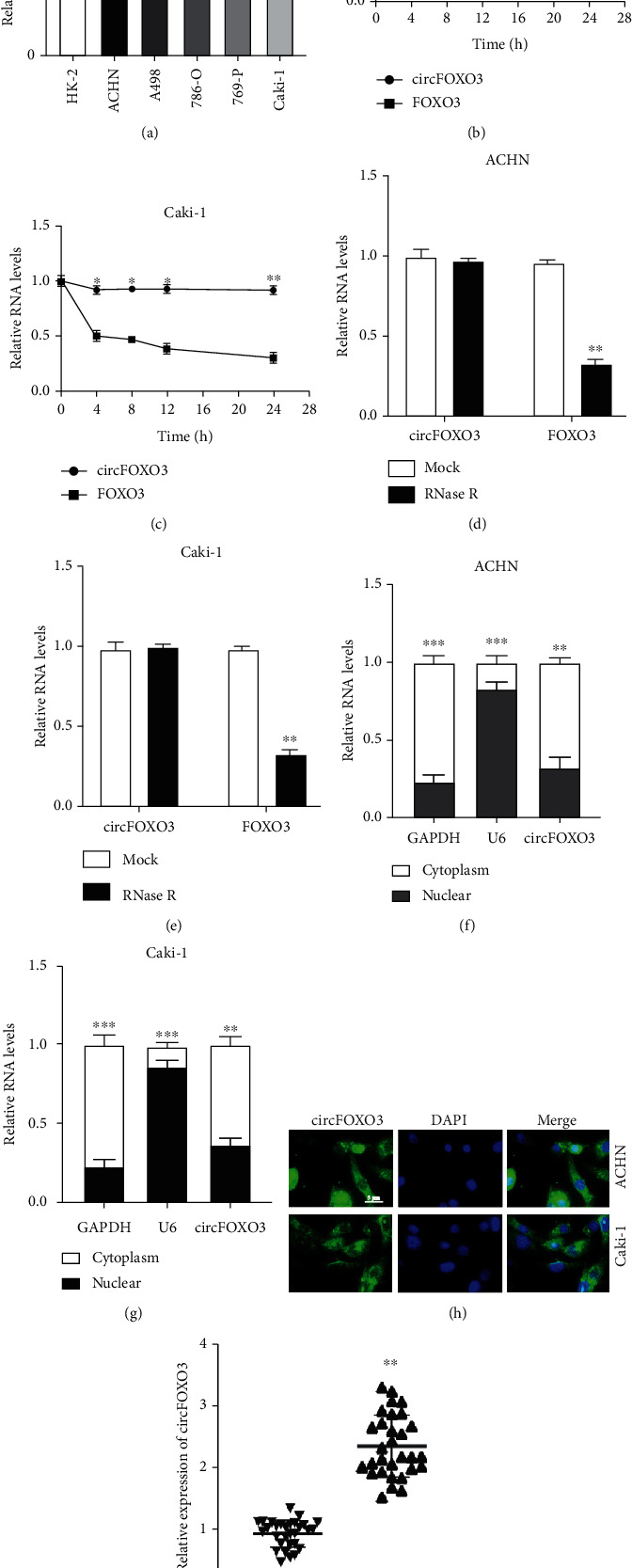
Expression and characterization of circFOXO3 in ccRCC. (a) qRT-PCR was conducted to quantify circFOXO3 expression in ccRCC cell lines. Expression of circFOXO3 or FOXO3 in (b) ACHN or (c) Caki-1 after actinomycin D treatment. Expression of circFOXO3 or FOXO3 in (d) ACHN or (e) Caki-1 after RNase treatment. circFOXO3 distribution in (f) ACHN or (g) Caki-1. (h) RNA-FISH assay to locate circFOXO3 expression in ACHN or Caki-1 cells. (i) Expression of circFOXO3 in thirty pairs of ccRCC tumor and adjacent normal tissues was measured by qRT-PCR (*n* = 3; ^∗^*P* < 0.05, ^∗∗^*P* < 0.01, and ^∗∗∗^*P* < 0.001).

To investigate whether circFOXO3 plays a role in ccRCC progression, the expression of circFOXO3 was examined in ccRCC cell lines and HK-2 cells, showing that circFOXO3 was generally upregulated in ccRCC cells (Figure 1(a)). To confirm the circular RNA characteristics of circFOXO3 in ccRCC cells, circFOXO3 and its linear form FOXO3 were measured in ACHN or Caki-1 cells after actinomycin D treatment, showing that circFOXO3 was more stable than its linear form after actinomycin D treatment (Figures 1(b) and 1(c)). Next, RNase R treatment was applied to digest RNAs with circFOXO3 being significantly more resistant to RNase R digestion compared to its linear form (Figures 1(d) and 1(e)). The intracellular distribution assay (Figures 1(f) and 1(g)) and RNA-FISH assay (Figure 1(h)) revealed that circFOXO3 was mainly located in the cytoplasm of ccRCC cells. Furthermore, circFOXO3 expression was upregulated in the thirty pairs of ccRCC tumor tissues compared to their adjacent normal tissues (Figure 1(i)) indicating that circFOXO3 might exert a biological function in ccRCC progression.

### 3.2. Dysregulation of circFOXO3 Influences the Susceptibility of ccRCC Cells to NK Cells

**Figure 2:**
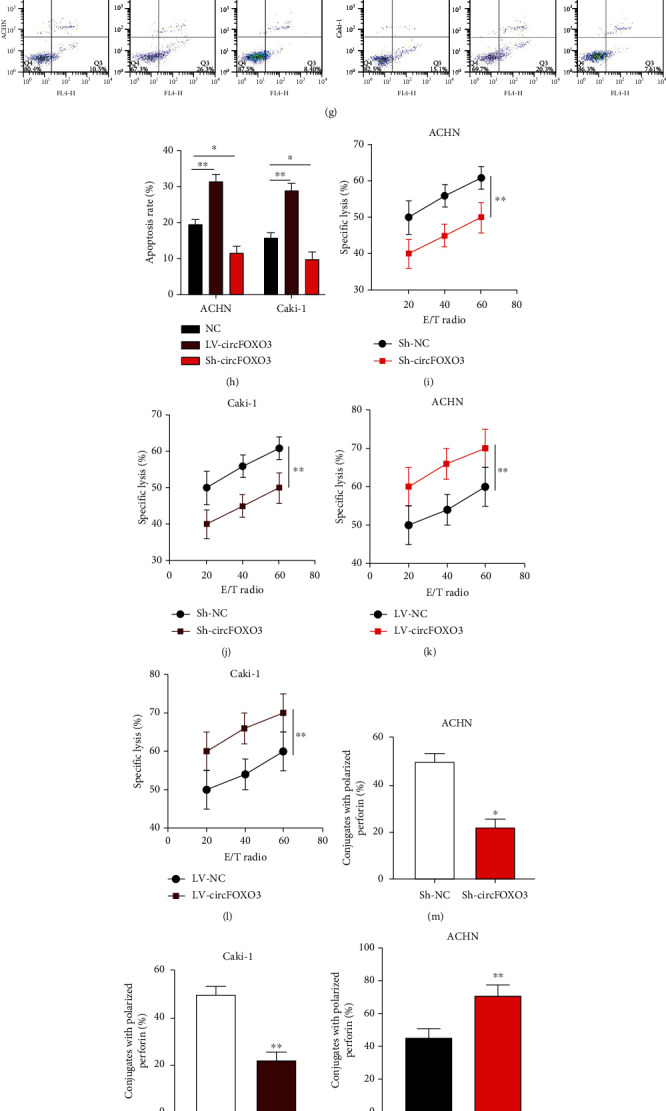
Dysregulation of circFOXO3 influences the susceptibility of ccRCC cells to NK cells. (a–d) ACHN and Caki-1 cells were stably transfected with Sh-NC, Sh-circFOXO3, LV-NC, or LV-circFOXO3 as indicated before circFOXO3 expression was quantified by qRT-PCR. (e, f) The proliferation of transfected ACHN and Caki-1 cells was assessed by EdU incorporation. (g, h) Apoptosis of transfected ACHN and Caki-1 cells was measured by flow cytometry. (i–l) Calcein release assay to evaluate the NK cell cytotoxicity towards ACHN and Caki-1 cells. (m–p) Analysis of conjugated polarized perforin NK cells. (q–t) Conjugation of NK cells and infected ACHN or Caki-1 cells (*n* = 3; ^∗^*P* < 0.05 and ^∗∗^*P* < 0.01).

As an antitumor innate immune factor, NK cells are an essential tumor suppressor in tumorigenesis, including ccRCC [27, 28], but whether circFOXO3 influences the susceptibility of ccRCC cells towards NK cells is unknown. circFOXO3 knockdown or overexpression cell models were constructed (Figures 2(a)–2(d)), showing that circFOXO3 overexpression inhibited the proliferation and promoted cell apoptosis of ccRCC cells, which would be reversed when circFOXO3 was downregulated (Figures 2(e)–2(h)). Interestingly, the calcein release assay (Figures 2(i)–2(l)), perforin polarization assay (Figures 2(m)–2(p)), and conjugation assay (Figures 2(q)–2(t)) revealed that circFOXO3 overexpression significantly promoted cell death, whereas circFOXO3 downregulation markedly inhibited cell death, indicating that circFOXO3 aggravates the cytotoxic activity of NK cells to ccRCC cells.

### 3.3. circFOXO3 Directly Sponges miR-29a-3p and miR-122-5p

**Figure 3:**
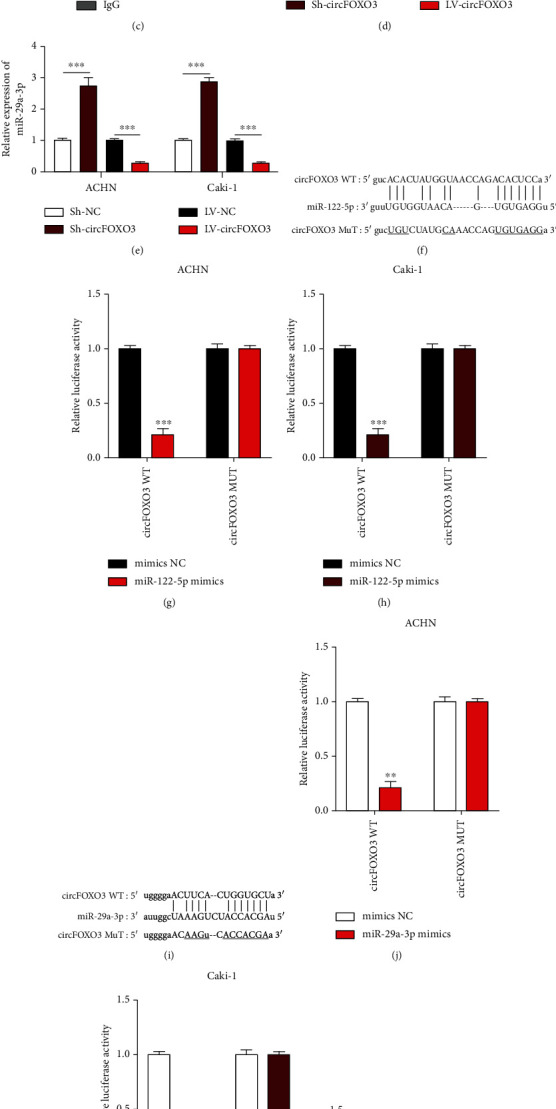
circFOXO3 directly sponges miR-29a-3p and miR-122-5p. Bioinformatics analysis using the Starbase 2.0v dataset (http://starbase.sysu.edu.cn/, CLIP data: strict stringency (≥5), class: 8mer) identified seven putative miRNAs (miR-5586-5p, miR-409-5p, miR-29a-3p, miR-122-5p, miR-6088, miR-29c-3p, and miR-29b-3p). RNA pull-down assays using biotinylated probes to evaluate putative sponging targets of circFOXO3 in (a) ACHN and (b) Caki-1 cells. (c) A RIP assay using anti-AGO2 or anti-IgG antibodies was conducted to assess the miRNA binding abilities of circFOXO3 in ACHN or Caki-1 cells. Relative expression level of (d) miR-29a-3p or (e) miR-122-5p in circFOXO3 dysregulated ACHN or Caki-1 cells quantified by qRT-PCR. (f) Bioinformatic prediction of the putative binding sites of circFOXO3 and miR-122-5p. The interaction between circFOXO3 and miR-122-5p was confirmed by a luciferase reporter assay in (g) ACHN and (h) Caki-1 cells. (i) The putative binding sites between circFOXO3 and miR-29a-3p. The interaction between circFOXO3 and miR-29a-3p was confirmed by luciferase reporter assay in (j) ACHN and (k) Caki-1 cells. Expression of (l) miR-122-5p or (m) miR-29a-3p in thirty pairs of ccRCC tissues (*n* = 3; ^∗^*P* < 0.05, ^∗∗^*P* < 0.01, and ^∗∗∗^*P* < 0.001).

Seven putative miRNA targets of circFOXO3 were identified by bioinformatics analysis for an RNA pull-down assay with biotinylated RNA probes to assess the interaction with circFOXO3, showing that miR-29a-3p and miR-122-5p were abundantly enriched in bio-circFOXO3 probes compared to bio-NC probes in ACHN (Figure 3(a)) or Caki-1 (Figure 3(b)) cells. The possible miRNA binding ability of circFOXO3 in ACHN or Caki-1 cells was detected by RIP assay with Argonaute RISC Catalytic Component 2 (AGO2) antibody (Figure 3(c)) revealing that circFOXO3 negatively regulated miR-122-5p or miR-29a-3p in ACHN or Caki-1 cells (Figures 3(d) and 3(e)). To confirm the interaction between circFOXO3 and miR-122-5p or miR-29a-3p, predicted binding sites of circFOXO3 and miR-122-5p (Figure 3(f)) or miR-29a-3p (Figure 3(i)) were synthesized and the association between circFOXO3 and miR-122-5p (Figures 3(g) and 3(h)) or miR-29a-3p (Figures 3(j) and 3(k)) was assessed by dual-luciferase reporter assay. The results indicated that circFOXO3 directly targets miR-122-5p or miR-29a-3p in ccRCC cells. Moreover, miR-122-5p or miR-29a-3p expression was downregulated in thirty pairs of ccRCC tumor tissues compared to normal tissues (Figures 3(l) and 3(m)).

### 3.4. Upregulation of miR-29a-3p or miR-122-5p Attenuates the NK Cell-Mediated Cytotoxicity to ccRCC Cells

**Figure 4:**
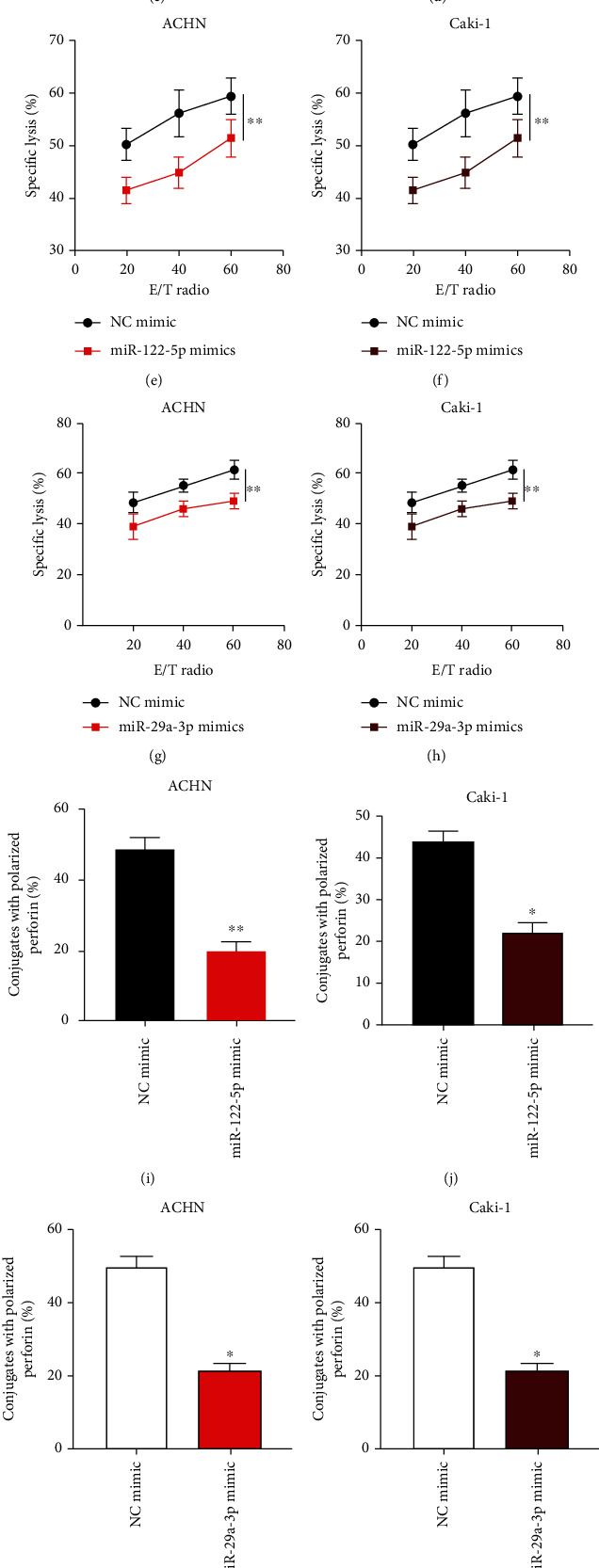
Upregulation of miR-29a-3p or miR-122-5p attenuates the NK cell-mediated cytotoxicity to ccRCC cells. (a–d) ACHN and Caki-1 cells were infected with NC mimic, miR-122-5p mimic, or miR-29a-3p mimic as indicated, and relative miRNA expression was assessed by qRT-PCR. (e–h) Calcein release assay to evaluate the NK cell cytotoxicity towards ACHN and Caki-1 cells as indicated. (i–l) Quantification of conjugated polarized perforin NK cells. (m–p) Conjugation assay to assess the formation of NK cells and infected ACHN or Caki-1 cell conjugates (*n* = 3; ^∗^*P* < 0.05, ^∗∗^*P* < 0.01, and ^∗∗∗^*P* < 0.001).

miRNA overexpression cell models were generated as indicated in Figures 4(a)–4(d). The calcein release assay (Figures 4(e)–4(h)) showed that ACHN or Caki-1 cell death was suppressed by miR-122-5p or miR-29a-3p overexpression. Subsequently, the perforin polarization results (Figures 4(i)–4(l)) suggested that upregulated miR-122-5p or miR-29a-3p significantly inhibited NK cell polarized conjugate formation in ACHN or Caki-1 cells. Furthermore, NK cell conjugate formation was reduced by miR-122-5p or miR-29a-3p overexpression in ACHN or Caki-1 cells (Figures 4(m)–4(p)). Taken together, these results suggest that the NK cell-mediated cytotoxicity towards ccRCC cells could be attenuated by miR-122-5p or miR-29a-3p overexpression.

### 3.5. circFOXO3 Regulates the NK Cell-Mediated Cytotoxicity to ccRCC Cells via Interacting with miR-29a-3p and miR-122-5p

**Figure 5:**
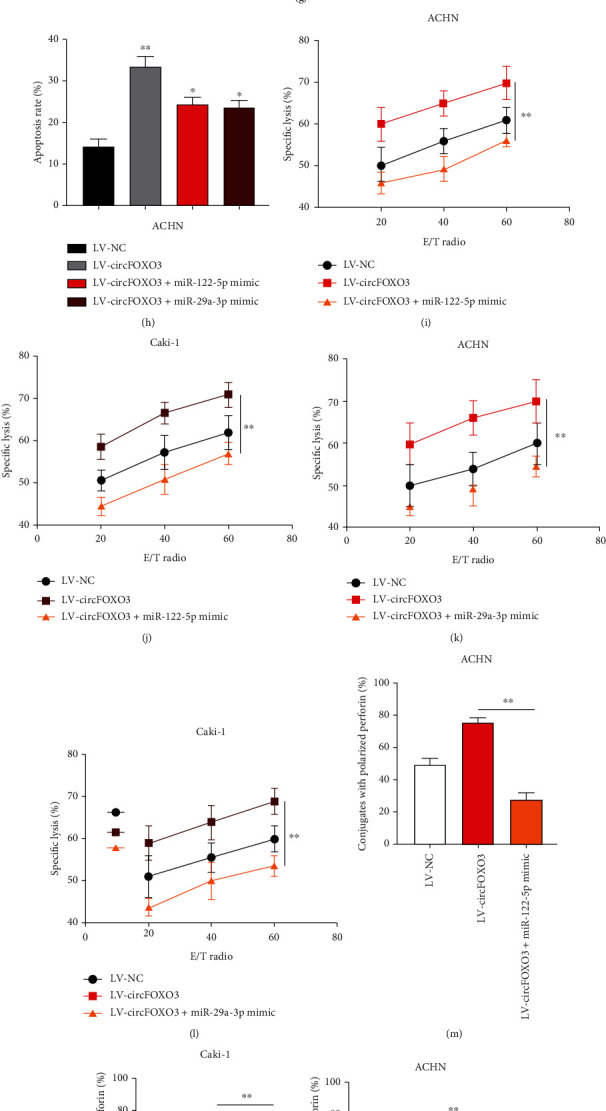
circFOXO3 regulates the NK cell-mediated cytotoxicity towards ccRCC cells via interacting with miR-29a-3p and miR-122-5p. (a–d) Cell models were constructed by infecting LV-NC, LV-circFOXO3, LV-circFOXO3+miR-122-5p mimic, or LV-circFOXO3+miR-29a-3p mimic into ACHN or Caki-1 cells as indicated, and infection efficiencies were determined by qRT-PCR. (e, f) The proliferation of transfected ACHN cells was measured by EdU. (g, h) Apoptosis of transfected ACHN cells was assessed by flow cytometry. (i–l) Calcein release assay to evaluate NK cell cytotoxicity towards ACHN and Caki-1 cells as indicated. (m–p) Quantification of conjugated polarized perforin NK cells. (q–t) Conjugation assay to assess the conjugate formation of NK cells and infected ACHN or Caki-1 cell conjugates (*n* = 3; ^∗^*P* < 0.05, ^∗∗^*P* < 0.01, and ^∗∗∗^*P* < 0.001).

Cell models were constructed to determine the function of the circFOXO3/miR-122-5p/miR-29a-3p axis in ccRCC progression as shown in Figures 5(a)–5(d). It was found that the inhibitory role of circFOXO3 on cell proliferation was partially attenuated by miR-122-5p or miR-29a-3p (Figures 5(e) and 5(f)). Furthermore, miR-122-5p or miR-29a-3p could partially alleviate the promotive effect of circFOXO3 on cell apoptosis (Figures 5(g) and 5(h)). The calcein release assay (Figures 5(i)–5(l)), perforin polarization assay (Figures 5(m)–5(p)), and conjugation assay (Figures 5(q)–5(t)) indicated that the promotive effect of circFOXO3 overexpression on ACHN or Caki-1 cell death was rescued by miR-122-5p or miR-29a-3p overexpression, partially verifying the biological function of circFOXO3/miR-122-5p/miR-29a-3p in ccRCC development.

### 3.6. circFOXO3 Expression Is Transcriptionally Regulated by KLF16

**Figure 6:**
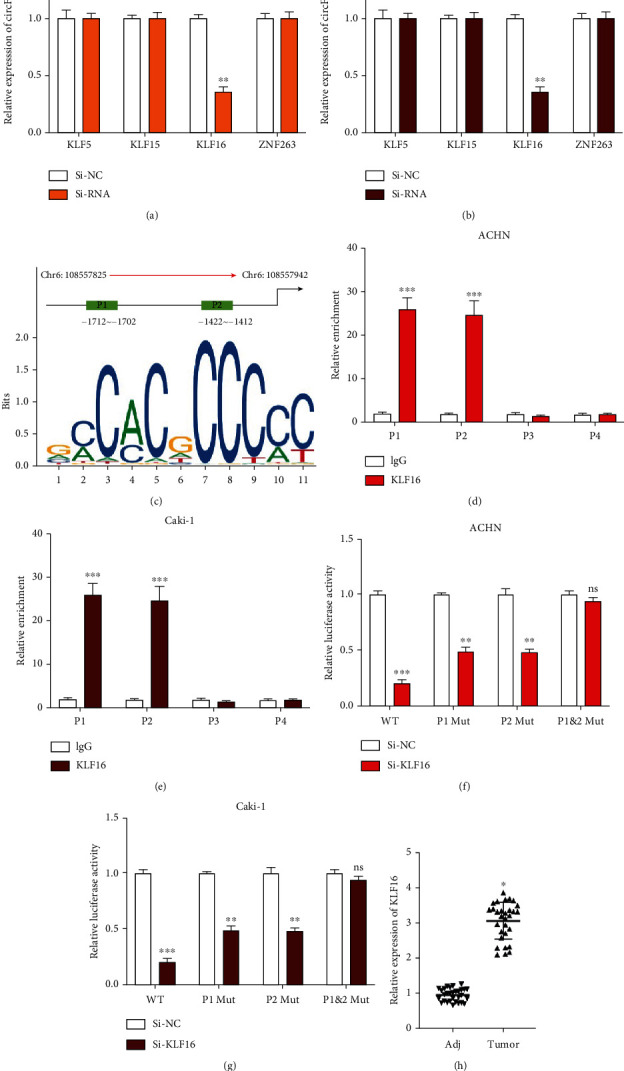
circFOXO3 expression is transcriptionally regulated by KLF16. The upstream transcriptional regulators of circFOXO3 were predicted using NCBI (https://www.ncbi.nlm.nih.gov/), UCSC (http://genome.ucsc.edu/), and JASPAR (http://jaspar.genereg.net/) datasets. Relative expression of circFOXO3 in (a) ACHN or (b) Caki-1 cells pretransfected with the indicated siRNAs quantified by qRT-PCR. (c) Binding sites of the FOXO3 promoter and KLF16 were predicted by the JASPAR dataset (relative profile score threshold 95%). Relative enrichment of FOXO3 promoter regions (P1-P4) in anti-IgG or anti-KLF16 bounds was analyzed by qRT-PCR in (d) ACHN or (e) Caki-1 cells using the IgG group as the control. (f, g) The interaction between KLF16 and FOXO3 promoter (P1/P2) regions was assessed by a luciferase reporter gene assay. (h) Expression of KLF16 in thirty pairs of ccRCC tissues measured by qRT-PCR (*n* = 3; ^∗^*P* < 0.05, ^∗∗^*P* < 0.01, and ^∗∗∗^*P* < 0.001).

circRNA expression is regulated by transcription factors [29, 30], so the JASPAR tool was used to predict four upstream transcription regulators of the circFOXO3 promoter. Subsequently, circFOXO3 expression was shown to be reduced by si-KLF16 in ACHN (Figure 6(a)) or Caki-1 (Figure 6(b)) cells suggesting that KLF16 might be the upstream regulator of circFOXO3. Next, the predicted binding region of KLF16 and the circFOXO3 promoter was obtained from the JASPAR database (Figure 6(c)). The P1 and P2 regions of FOXO3 promoter could be enriched in anti-KLF16 bounds in ACHN (Figure 6(d)) or Caki-1 (Figure 6(e)) cells, indicating that KLF16 might interact with the P1 and P2 regions of the circFOXO3 promoter. The possible interaction was confirmed by a dual-luciferase reporter assay, showing that KLF16 knockdown markedly reduced the luciferase activity of the WT circFOXO3 promoter, which was alleviated when the circFOXO3 promoter was mutated (P1 or P2 Mut) in the P1 and P2 regions (Figures 6(f) and 6(g)). KLF16 expression in ccRCC tumor tissues was upregulated compared to that in adjacent normal tissues (Figure 6(h)) suggesting that circFOXO3 expression in ccRCC is transcriptionally upregulated by KLF16.

## 4. Discussion

ccRCC is one of the most lethal cancer types characterized by high incidence, recurrence, and mortality rates [31]. Furthermore, nearly one-third of ccRCC patients are diagnosed at a localized or distant metastatic status [32, 33], and despite advancements in medication management or surgical intervention, the five-year survival rate of ccRCC is still less than 10% [34]. For surgical intervention, nearly 40% of patients who undergo partial or radical nephrectomy experienced cancer recurrence and only 20% survived for five years [35]. Moreover, ccRCC patients are resistant to chemotherapy or radiotherapy [36]. Immunotherapy for RCC is rapidly expanding globally and has significantly improved clinical outcomes for RCC patients, but novel therapeutic targets for ccRCC clinical intervention are urgently needed [37].

This study investigated the role of circFOXO3 in RCC, confirming that circFOXO3 is abundantly expressed in ccRCC tissues and cells, predominately located in the cell cytoplasm, and thus may be related to ccRCC progression. Next, we verified that circFOXO3 inhibited the viability of ccRCC cells. NK cells are a well-known antitumor innate immune factor and an essential tumor suppressor in various cancers including ccRCC [27, 28]. Although the function of circFOXO3 in multiple tumors has been elucidated, whether circFOXO3 influences NK cell susceptibility remains unknown. Of interest, we explored the biological function of circFOXO3 in the interaction of ccRCC cells and NK cells, showing that dysregulation of circFOXO3 modulates NK cell toxicity towards ccRCC cells. It has been well documented that NK cells play a crucial role in the human immune system, such as secreting cytokines, killing cancer cells directly, and suppressing tumor cell growth or metastasis [38, 39]. Hence, our result might enrich the prognostic value of circFOXO3 in ccRCC. Subsequent molecular analysis revealed that circFOXO3 sponges miR-29a-3p and miR-122-5p and that overexpression of miR-29a-3p or miR-122-5p attenuated NK cell toxicity to ccRCC cells.

The expression of circRNAs can be regulated by transcription factors; for example, circSEPT9 expression in triple-negative breast cancer cells is mediated by E2F1 and EIF4A3 [40]. In glioma stem cells, circRNA ARF1 is transcriptionally regulated by U2AF2 [41] and circRNF121 in osteoarthritis is regulated by LEF1 [42]. Herein, the JASPAR dataset was utilized to show that circFOXO3 expression was transcriptionally regulated by KLF16 in ccRCC cells.

The present study has partially elucidated the biological function of circFOXO3 in ccRCC progression, but a more in-depth investigation using clinical samples is required to confirm the results, as well as to further investigate the KLF16/circFOXO3/miR-29a-3p/miR-122-5p axis. Nonetheless, this novel KLF16/circFOXO3/miR-29a-3p/miR-122-5p axis in ccRCC progression may help to identify novel diagnostic or therapeutic targets for ccRCC in the future.

## Data Availability

All data are available from the corresponding author upon reasonable request.
